# Immunoglobulin heavy/light chain analysis enhances the detection of residual disease and monitoring of multiple myeloma patients

**DOI:** 10.3325/cmj.2015.56.263

**Published:** 2015-06

**Authors:** Josip Batinić, Zinaida Perić, Dragana Šegulja, James Last, Sanja Prijić, Klara Dubravčić, Lidija Volarić, Dubravka Sertić, Ivo Radman, Sandra Bašić-Kinda, Danica Matišić, Drago Batinić, Boris Labar, Damir Nemet

**Affiliations:** 1 School of Medicine, University of Zagreb, Zagreb, Croatia; 2 University Hospital Center Zagreb, Department of Internal Medicine, Division of Hematology, Zagreb, Croatia; 3 University Hospital Center Zagreb, Department of Laboratory Diagnostics, Clinical Unit of Special Biochemistry, Zagreb, Croatia; 4 University Hospital Center Zagreb, Department of Laboratory Diagnostics, Clinical Unit of Cellular Immunodiagnostics and In Vitro Procedures, Zagreb, Croatia; 5 The Binding Site Group Ltd, Birmingham, UK

## Abstract

**Aim:**

To evaluate the clinical utility of incorporating a novel heavy/light chain immunoassay (HLC) into the existing methods for the assessment of multiple myeloma (MM) patients.

**Methods:**

Convenience sera samples from 90 previously treated IgG and IgA MM patients in different disease stages were analyzed. The study was conducted in Clinical Hospital Center Zagreb between 2011 and 2013. The collected sera were analyzed by standard laboratory techniques (serum protein electrophoresis, quantification of total immunoglobulins, serum immunofixation, serum free light chain [FLC] assay) and HLC assay.

**Results:**

HLC ratios outside the normal range were found in 58 of 90 patients, including 28 out of 61 patients with total immunoglobulin measurements within the normal range and 5 out of 23 patients in complete response. Both elevated HLC isotype level and abnormal HLC ratio correlated with the parameters of tumor burden, including percentage of plasma cells in the bone marrow (*P* < 0.001 and *P* = 0.002, respectively) and an abnormal serum FLC ratio (for both *P* < 0.001). In addition, abnormal HLC isotype level correlated with serum beta-2-microglobulin level (*P* = 0.038). In terms of prognosis, abnormal HLC isotype level and abnormal HLC ratio were significantly associated with shorter overall survival (*P* < 0.001 and *P* = 0.002, respectively). Interestingly, suppression of the uninvolved (polyclonal) isotype pair, but not other non-myeloma immunoglobulin isotypes, was also associated with a shorter overall survival (*P* = 0.021). In a multivariate analysis, an abnormal HLC ratio and β_2_-microglobulin level >3.5mg/L were independent risk factors for survival.

**Conclusion:**

The new HLC assay has greater sensitivity in detecting monoclonal protein, correlates with tumor burden markers, and affects patients' outcome.

Hematological malignancy multiple myeloma is characterized by the proliferation of monoclonal plasma cells in the bone marrow, which usually leads to the secretion of monoclonal intact immunoglobulins (Ig), free light chains (FLC), or both, into the serum. Consequently, the measurement of monoclonal proteins is integral to diagnosing multiple myeloma, monitoring of response to treatment, and detecting relapse (1-3). Conventional methods rely principally on serum electrophoresis together with either urine electrophoresis for the detection of FLC M-proteins or nephelometric quantification of total Igs for intact Ig paraprotein measurement. However, these standard techniques often do not have the required sensitivity or do not correlate with the patient’s clinical status (4-7).

The ability to detect monoclonal serum FLC has been substantially increased by the introduction of the serum FLC assay, improving the diagnosis, monitoring, and prognosis of a number of plasma cell dyscrasias (8,9). Still, a number of problems associated with the detection of monoclonal intact Igs persist. Serum Ig concentrations can change for ≥50% with fluctuations in blood volume and/or hematocrit (10). In the case of monoclonal IgGs, the serum concentration can be also influenced by altered metabolism due to recycling of IgG by the neonatal Fc receptors (11-13). Furthermore, monoclonal proteins, in particular IgA paraproteins, may not be accurately quantified by serum protein electrophoresis (SPE) due to co-migration with other serum proteins (14,15). Finally, one of the major limitations of measuring total Igs is the inability to distinguish between the monoclonal component and polyclonal Ig background.

These problems can be addressed by a recently developed heavy/light chain (HLC) immunoassay. Using specific antibodies targeted to junctional epitopes of the heavy and light chains of Ig molecules, HLC immunoassay separately quantifies different light chain types of each Ig class (IgGκ, IgGλ, IgAκ, IgAλ, etc), thus providing accurate quantification of both the involved and uninvolved components of the patient's affected Ig isotype. The ratio of the monoclonal and polyclonal Igs of the same isotype (IgGκ/IgGλ, IgAκ/IgAλ) provides a sensitive measure of monoclonality, while minimizing the influence of changes in blood volume/hematocrit and the effect of IgG recycling (16,17).

Previous studies using this novel assay in specific, controlled patient cohorts found the HLC ratio to be useful for screening, monitoring, and risk stratification of patients with multiple myeloma, but also with other monoclonal gammopathies, such as monoclonal gammopathy of undetermined significance (MGUS) and amyloid light-chain (AL) amyloidosis (18-20). The aim of this study was to evaluate the ability of the new assay – HLC isotype measurements – to detect monoclonal protein (ie, residual disease) in previously treated multiple myeloma patients and to determine whether these patients can benefit from adding the new assay into a routine assessment.

## Patients and methods

The study included 90 unselected intact Ig multiple myeloma patients in different clinical stages (symptomatic disease) who had received at least one line of different treatment regiments (the number of patients was limited by availability of serum assays but also by the number of patients who were willing to enter the study). Written informed consent was obtained from each patient before entering the study. Patients were enrolled consecutively as they presented for routine assessment at our hospital between January 2011 and October 2013. At the time of the assessment, all patients already completed treatment regiments. The median follow-up period was 17.4 months (range 0.5-46.1 months; follow up period is defined as time elapsed from the day of the sampling until January 2015 when statistical analysis was performed). Median age was 61 years (range 39-84). A complete clinical and laboratory assessment was performed on convenience samples using standard laboratory techniques (SPE, nephelometric quantification of total immunoglobulins, serum immunofixation [IFE], and serum FLC assay) in addition to the novel HLC assay, complete blood count, and bone marrow analysis. Biochemical parameters – serum albumin, β_2_-microglobulin, serum creatinine, serum calcium levels, and lactate dehydrogenase were also measured. This study was approved by the Ethics Committee of the University Hospital Center Zagreb and Ethics Committee of the Medical School, University of Zagreb.

Serum HLC and FLC levels were measured using the polyclonal antisera assays Hevylite® and Freelite®, respectively (The Binding Site, Birmingham, UK) and performed on a Dade Behring BNII analyzer (Siemens AG, Munich, Germany) according to the manufacturer's instructions. Normal ranges for HLC isotypes and their ratios were obtained from the manufacturer's instructions. SPE and sIFE were performed on a Hydrasys platform (Sebia, Paris, France). Total Igs were measured using Tina-quant Gen.2 reagents (Roche, Rotkreuz, Switzerland) on a Cobas® 6000 platform (Roche) according to the manufacturer's instructions.

The data were analyzed using the R package (21). Subgroups of patients were compared using the Mann-Whitney test for continuous variables and the χ^2^ for categorical variables. Overall survival (OS) was estimated with the Kaplan-Meier method and the subgroups of patients were compared using the log-rank test. The association of time to death with relevant variables was evaluated in a multivariate analysis with the use of Cox’s proportional-hazard regression model. *P* values ≤0.05 were considered significant.

## Results

A total of 66/90 (73%) patients had IgG myeloma (47 IgGκ and 19 IgGλ) and 24/90 (27%) had IgA myeloma (13 IgAκ and 11 IgAλ). According to international response criteria (22), at the moment of the study 67/90 patients had achieved a partial response or better (10 partial responses, 34 very good partial responses, and 23 complete responses), 1 patient showed minimal response, 10 patients had stable disease, 7 patients had progressive disease, and 5 patients were at relapse (Table 1).

**Table 1:** Study population characteristics at re-evaluation. Values are shown as medians and range

	All	IgG patients	IgA patients
Number of patients	90	66	24
Age (years)	61 (39-84)	61 (39-84)	64 (45-84)
Sex (male/female)	42/48	32/34	10/14
Total immunoglobulin – standard method (g/L)	10.48 (1.05-54.32)	11.48 (2.85-54.32)	4.76 (1.05-35.49)
Serum free light chain ratio	1.08 (0.0-299.0)	1.16 (0.0-299.0)	0.75 (0-25.8)
β-2-microglobulin (mg/L)	2.34 (1.3-11.15)	2.3 (1.3-11.15)	2.45 (1.70-9.78)
Albumin (g/L)	41.8 (16.8-69.0)	42.15 (30.6-69.0)	39.2 (16.8-44.9)
Hemoglobin (g/L)	125 (86-179)	125 (86-149)	125 (101-179)
Thrombocytes ( × 10^9^/L)	187 (43-567)	190 (43-567)	184 (99-378)
Creatinine (µmol/L)	90 (47-256)	90 (47-172)	77 (51-256)
Serum calcium (mmol/L)	2.34 (1.82-2.86)	2.33 (1.97-2.79)	2.43 (1.82-2.86)
Lactate dehydrogenase (U/L)	182 (79-862)	190 (79-862)	158 (113-264)
Percentage of plasma cells	6 (0-95)	6 (0-70)	7.5 (0-95)
International Staging System stage I / II / III (No.)	53 / 34 / 3	36 / 29 / 1	17 / 5/ 2
Complete response (No.)	23	15	8
Very good partial response (No.)	34	28	6
Partial response (No.)	10	8	2
MR/SD/PD/relapse (No.)	1 /10 / 7 / 5	1 / 5 / 4 / 5	0 / 5 / 3 / 0

*MR – minimal response; SD – stable disease; PD – progressive disease.

### The ability of the new assay to detect monoclonal immunoglobulin

One of the aims of our study was to determine the ability of HLC isotype measurement to detect monoclonal protein and to compare it with the standard test for Ig measurement. Standard test showed abnormal (elevated) total Ig measurement in 29/90 (32%) patients and Ig measurement within the normal range in 61/90 (68%) of patients. All patients with abnormal total Ig values according to standard test also had abnormal Ig values according to the new assay (abnormal involved HLC isotype value and/or abnormal HLC ratio). However, 28 (46%) of the 61 patients with normal Ig values according to the standard test (28/90 of total, 31%) had an abnormal HLC ratio, signifying the presence of monoclonal protein.

### Relationship between HLC ratio and serum FLC ratio

Abnormal HLC ratio in the presence of normal FLC ratio revealed monoclonal protein in additional 4/23 (17%) patients in complete response, 8/34 (24%) patients with very good partial response, and 3/10 (30%) patients with stable disease (Table 2). In all patients with progressive disease, both assays were positive. HLC and FLC assays provided sometimes differing but complementary information, ie, in some patients both assays indicated the presence of monoclonal protein and in others only one of the assays did so. This was perhaps most notable in 8/23 patients who had achieved complete response (Table 2). Nevertheless, 2 of the 5 patients in complete response but with an abnormal HLC ratio suffered a relapse during the follow up period.

**Table 2:** Results of **h**eavy/light chain (HLC) and free light chain (FLC) assays in multiple myeloma patients in different disease stages

	Number of patients with:			
Results	complete response (N = 23)	very good partial response (N = 34)	partial response (N = 10)	stable disease (N = 10)
FLC κ/λ ratio* and HLC Ig´κ/Ig´λ ratio both normal	14	12	0	0
FLC κ/λ ratio and HLC Ig´κ/Ig´λ ratio both abnormal	1	12	10	6
FLC κ/λ ratio abnormal and HLC Ig´κ/Ig´λ ratio normal	4	2	0	1
FLC κ/λ ratio normal and HLC Ig´κ/Ig´λ ratio abnormal	4	8	0	3

*free κ/λ light chain ratio.

†heavy/light Ig’κ/Ig’λ chain ratio.

### Disease variables and involved (monoclonal) HLC measurements

In order to assess whether the new assay truly reflected disease activity and provided good measurement of tumor burden (ie, residual disease), we tested the correlations between HLC measurements (involved HLC isotype values and HLC ratio) and other disease variables. Looking at the entire cohort, abnormal HLC ratio values correlated with the percentage of clonal plasma cells in the bone marrow (*P* = 0.002) and an abnormal serum FLC ratio (*P* < 0.001). Involved HLC isotype concentration above normal values correlated with serum beta-2-microglobulin values (*P* = 0.038), percentage of clonal plasma cells in the bone marrow (*P* < 0.001), and abnormal serum FLC ratio (*P* < 0.001).

Analyzing the data according to Ig isotype, abnormal IgGκ/IgGλ HLC ratios (normal range 0.98-2.75) correlated with the percentage of clonal plasma cells in the bone marrow (*P* = 0.020), abnormal serum FLC ratio (*P* < 0.001), and hemoglobin concentration (*P* = 0.032). Monoclonal IgGκ or IgGλ isotype concentrations above the normal upper limit (for IgGκ above 9.78 g/L and for IgGλ above 5.71 g/L) correlated with serum beta-2-microglobulin concentration (*P* = 0.011), percentage of clonal plasma cells in the bone marrow (*P* = 0.007), and abnormal serum FLC ratio (*P* = 0.002).

Abnormal IgAκ/IgAλ ratios (normal range 0.80-2.04) correlated with abnormal serum FLC ratio (normal range 0.26-1.65; *P* = 0.023) and serum beta-2-microglobulin concentration (*P* = 0.04), while correlation with the percentage of clonal plasma cells in the bone marrow was not as strong (*P* = 0.08). Monoclonal IgAκ or IgAλ isotype concentrations higher than normal upper limit (for IgAκ above 2.82 g/L and for IgAλ above 1.98 g/L) correlated with serum beta-2-microglobulin concentration >3.5 mg/L (*P* = 0.050) and abnormal serum FLC ratio (*P* = 0.005).

### Disease variables and uninvolved (polyclonal) HLC isotype level

Correlations regarding the suppression of the polyclonal HLC isotype pair (ie, suppression of IgGλ in a IgGκ myeloma patient), were also assessed. Suppression was defined as an istoype pair value below normal range. In the entire group, there were significant correlations between isotype pair suppression and abnormal serum FLC ratio (*P* < 0.001), as well as hemoglobin concentration <100 g/L (*P* = 0.005). The same was found when looking at IgG group alone (abnormal serum FLC ratio, *P* < 0.001 and hemoglobin <100 g/L, *P* < 0.001), while in the IgA group there were no significant correlations between the suppressed polyclonal isotype and other tumor markers, possibly due to the small number of patients.

### HLC measurements and overall survival

**Figure 1:**
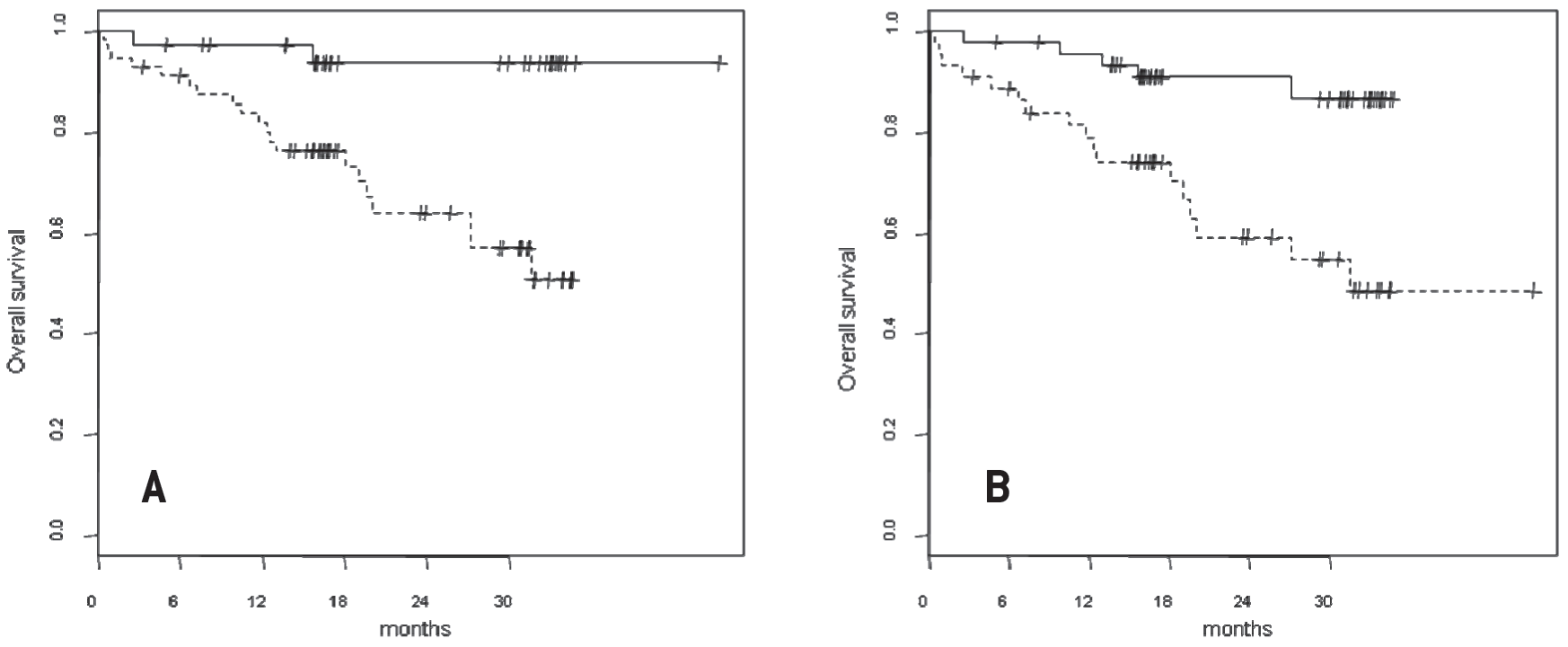
Overall survival in study population. (**A**) Patients stratified according to heavy/light chain ratio. Those with an abnormal value had significantly shorter overall survival (dotted line) than those with normal values (full line, *P* = 0.0019); median survival was not reached in either group. (**B**) Patients stratified according to monoclonal isotype absolute values. Median survival was 31.7 months in patients with monoclonal isotype values above normal (dotted line) and was not reached in patients with monoclonal isotype values with normal range (full line, *P* = 0.002).

The aim of this study was to determine not only the ability of the new assay to detect monoclonal protein, ie, residual disease but also the clinical relevance of HLC assay measurement in multiple myeloma patients regardless of the type of treatment and time elapsed from the last course of therapy. For this purpose, Kaplan-Meier analysis was performed for OS of patients from the date of enrolment onwards. Patients were stratified according to HLC ratio values, and OS was significantly shorter in patients with abnormal HLC ratio value (median OS not reached, *P* < 0.001) (Figure 1A). When patients were stratified according to monoclonal isotype concentrations, OS was significantly shorter in patients with values above normal range (median 31.7 months vs median not reached, *P* = 0.002) (Figure 1B).

**Figure 2:**
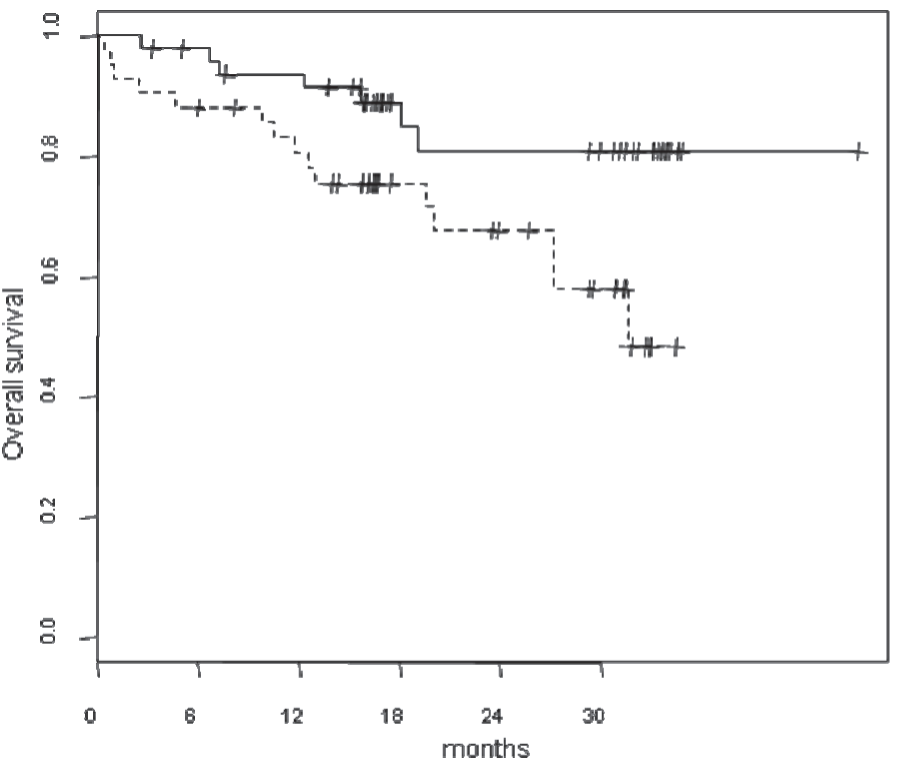
Overall survival in patients stratified according to polyclonal heavy/light chain (HLC) isotype pair suppression. Median overall survival was 31 months in patients showing suppressed polyclonal HLC isotypes (dotted line) and was not reached in patients without suppression (full line, *P* = 0.021).

Interestingly, when patients were stratified according to the presence or absence of suppression of the uninvolved polyclonal HLC isotype pair, those with isotype suppression also had significantly shorter OS (median 31 months vs median not reached, *P* = 0.021, Figure 2).

**Figure 3:**
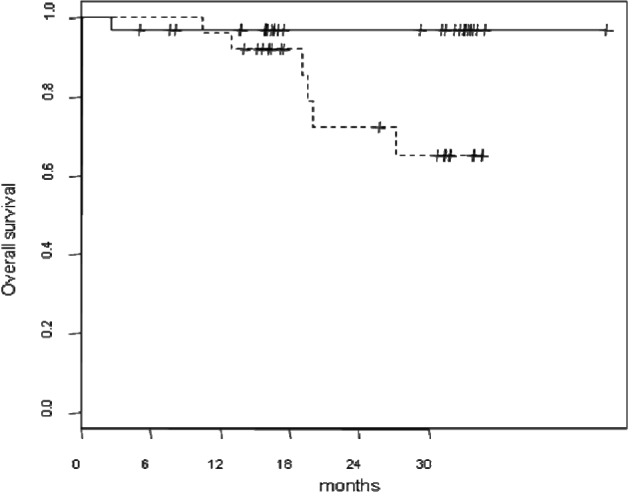
Overall survival in patients who achieved very good partial response/complete response stratified according to heavy/light chain ratio. Patients with an abnormal value had significantly shorter overall survival than those with normal values; median survival was 27.2 months (dotted line) vs not reached (full line, *P* = 0.002).

The clinical relevance of the HLC assay was even more evident when Kaplan-Meier analysis was performed in patients who achieved very good partial responses (defined as negative SPE and positive IFE) and complete response (both SPE and IFE negative). When this subgroup of patients was stratified according to HLC ratio value, OS was significantly shorter in patients with abnormal HLC ratio values than in very good partial responses/complete response patients with normal HLC ratio (median 27.2 month vs median not reached, *P* = 0.033) (Figure 3).

### Prognostic significance of HLC measurements

In order to assess the prognostic significance of the new assay, Cox multivariate regression analysis was performed for the entire study population and for the group of patients who achieved very good partial response or better. An abnormal HLC ratio as well as high beta-2-microglobulin (>3.5 mg/L) concentration were independent predictors of OS for the entire cohort (risk ratio [RR] 4.7, confidence interval [CI] 0.96-23.47, *P* = 0.050; RR 4.9, CI 1.59-15.34, *P* = 0.005, respectively) (Table 3). When assessing the patients with very good partial response or better, the significance of abnormal HLC ratio was not as strong as for the entire group (RR 3.18, CI 0.84-12.04, *P* = 0.087).

**Table 3:** Multivariate analysis showed abnormal heavy/light chain ratio to be an independent risk factor for overall survival

Risk factor	Relative risk	95% confidence interval	*P*
**β_2_-microglobulin >3.5 mg/L**	**4.94**	**1.59-15.34**	**0.005**
Abnormal heavy/light chain ratio*	**4.74**	**0.96-23.47**	**0.050**
Abnormal free light chain ratio	3.29	0.93-11.69	0.064
Serum albumin <35 g/L	2.18	0.67-7.06	0.191
International staging system (stage III)	0.71	0.13-3.90	0.699

*Variables in bold denote statistically significant results (*P* ≤ 0.05).

## Discussion

### Detection of monoclonal immunoglobulin

Using HLC assay, we successfully detected isotype and quantified monoclonal Ig in all multiple myeloma patients. HLC assay had greater sensitivity for monoclonal disease detection than total Ig result. It revealed an abnormal HLC ratio in almost half of the patients with total Ig levels within normal range, signifying the presence of monoclonal protein. Moreover, an abnormal HLC ratio was found in almost 20% of complete response patients, thus detecting the presence of residual disease when other laboratory techniques could not detect it. In support of this, 2 of the 5 patients in complete response but with an abnormal HLC ratio suffered relapse during the follow up period. Similar observations of abnormal HLC ratios in patients in complete response have been reported previously (18,23).

The HLC and serum FLC assays provided differing but complementary information. This was most apparent in patients in complete response: 4 patients had abnormal serum FLC ratio and normal HLC ratio, whereas 4 patients had the opposite results. Knowing the metabolism of FLC and intact Igs, one would expect that the normalization of the FLC ratio will precede or occur simultaneously with the normalization of HLC ratio. However, our data showed that normal HLC ratio values did not necessarily mean normal FLC ratio values or vice versa. Similar observations were reported previously (18) and are consistent with the two assays providing information on two independent markers of myeloma activity – the intact Igs and the FLC (24,25). These data suggest that both assays are required for the optimal follow-up of multiple myeloma patients. In support of this, when response of myeloma patients to treatment was assessed, it was shown that in patients achieving complete response or better, patients with abnormal both HLC and FLC ratio had a significantly shorter 2-year progression free survival (PFS) than those with either normal HLC or FLC ratio (26).

### Significance of the uninvolved HLC isotype pair suppression

Our results showing a strong association of suppression of the uninvolved HLC isotype pair with both markers of tumor burden (ie, FLC and hemoglobin) and shorter OS are particularly interesting. An analogous correlation of abnormal HLC ratios with shorter PFS in myeloma patients, particularly with more extreme HLC ratios, has been observed previously (27). Interestingly, this association has been shown to be driven primarily and specifically by suppression of the uninvolved polyclonal HLC isotype pair, while suppression of the non-tumor Igs of different isotypes showed no significant correlation with PFS. Similarly, HLC pair suppression has been shown to be an additional independent risk factor for progression to malignancy in MGUS patients alongside serum monoclonal protein size, heavy chain isotype, and an abnormal serum FLC ratio (20). The reason for the association of the outcome specifically with HLC pair-suppression in myeloma and MGUS remains unexplained. However, several studies in MGUS patients have noted higher levels of HLC pair suppression in IgG patients compared to IgA and IgM patients (20,28). An explanation for this is that due to relatively higher levels of background polyclonal IgG, which may help to prevent invasion of clonal plasma cells (29), suppression of normal IgG plasma cells may be a requirement for progression in IgG MGUS. Conversely, suppression of normal plasma cells may be less of a requirement for IgA MGUS progression and may help to explain the higher risk of progression shown by IgA MGUS patients (28). Interestingly, in multiple myeloma patients, we observed similar results of more frequent HLC pair suppression in IgG myeloma, with 35/66 (53%) IgG patients showing HLC pair suppression compared to 7/24 (29%) IgA patients, although the relative number of IgA patients was much lower. As multiple myeloma has been shown to be preceded consistently by MGUS (30,31), our data suggests that this possible differential requirement for isotype-specific pair suppression between IgG and IgA MGUS is maintained upon progression to multiple myeloma. Therefore, HLC pair suppression may be a useful marker of malignant disease, suggesting that it is useful to monitor the response of multiple myeloma patients to treatment.

### HLC measurement values and impact on patients’ outcome

Despite the fact that the data in this study were obtained from a heterogeneous group of multiple myeloma patients, we found a strong correlation between an abnormal HLC ratio and other markers of tumor burden (the percentage of clonal plasma cells in the bone marrow and an abnormal serum FLC ratio). Given the statistical strength of the correlation, one might expect this to impact the patient outcome. Indeed, patients with abnormal level of monoclonal HLC isotype or abnormal HLC ratio had significantly shorter OS than patients with normal monoclonal HLC isotype level or HLC ratio. In the group of patients who achieved very good partial response or better, HLC ratio value maintained its impact on patients' outcome while monoclonal HLC isotype concentrations above normal range did not.

### Prognostic significance of the HLC measurements

In order to further assess the clinical relevance of HLC assay in the management of previously treated multiple myeloma patients, we additionally performed multivariate Cox regression analysis for the entire group as well as for patients in very good partial response or better. The analysis for the entire cohort confirmed the prognostic relevance of the HLC ratio along with high β2-microglobulin values. Interestingly, the serum FLC ratio, which has been reported as predictive of outcome in several previous studies (32-35), was not significantly associated with OS in our study, most likely due to the fact that we did not include light chain multiple myeloma patients. The prognostic utility of HLC ratios in multiple myeloma using clinical presentation samples of untreated patients or patients recruited to clinical trials where all individuals received the same chemotherapeutic treatment followed by autologous stem cell transplantation (ASCT) treatment has recently been shown (18,27). However, in our study, patients were unselected and so were at different clinical stages of disease, received different chemotherapeutic regimes, and only a proportion had received ASCT treatment. Even in such a sample, we were able to evaluate the clinical utility of the HLC assay in everyday practice. Therefore, our finding that an abnormal HLC ratio, irrespective of disease stage and treatment regimen, is predictive of a poorer outcome in multiple myeloma is particularly noteworthy.

In conclusion, our results corroborate previous studies and show potential clinical utility of the HLC assay. In our study, the HLC ratio values correlated well with other prognostic tumor burden markers, showed enhanced sensitivity for the detection and quantification of monoclonal proteins, and affected patient outcome using a cohort of unselected patients in a daily practice setting. Interestingly, HLC pair suppression also correlated well with other tumor markers and was prognostic for shorter overall survival. Multivariate analysis confirmed an abnormal HLC ratio as an independent prognostic factor. Although larger studies using more defined groups of patients are required, our data suggest a clear role for HLC measurement in monitoring and response assessment of multiple myeloma patients with potential role in predicting patients’ outcome.
